# A Simple Model to Estimate the Increase in Pavement Life Due to the Traffic Wander for Application in Connected and Autonomous Vehicles

**DOI:** 10.3390/ma18112609

**Published:** 2025-06-03

**Authors:** Beata Gajewska, Marcin Gajewski, Jorge Pais, Liseane Thives

**Affiliations:** 1 Institute of Civil Engineering, Warsaw University of Life Sciences-SGGW, 02-776 Warsaw, Poland; 2 Faculty of Civil Engineering, Warsaw University of Technology, 00-637 Warsaw, Poland; marcin.gajewski@pw.edu.pl; 3 Department of Civil Engineering, University of Minho, 4800-058 Guimarães, Portugal; 4 Department of Civil Engineering, Federal University of Santa Catarina, Florianópolis 88040-900, Brazil; liseane.thives@ufsc.br

**Keywords:** road pavements, connected and autonomous vehicles, wander, pavement performance, model

## Abstract

The primary purpose of this paper is to investigate the impact of traffic wander on road pavement life for application in connected and autonomous vehicles. Research shows that in autonomous vehicles, drivers often follow the same path, leading to significant pavement damage on specific, well-defined paths. The paper examined the impact of traffic wander on pavement life by analysing two different wander distributions: normal and uniform. Based on the estimated pavement life for various pavement structures, a model that predicts the increase in pavement life due to traffic wander was developed for cracking and rutting prediction. The result of the research is the determination of relative pavement life influence functions, in which the variables are the traffic wander, asphalt layer thickness and subgrade stiffness. The obtained equations can be easily implemented for pavement service life extension evaluation. The model was also used to estimate the asphalt layer thickness as a function of the traffic expressed in terms of Equivalent Single Axle Load (ESALs). An analysis of the implications of the lateral distribution of traffic on the pavement thickness was presented. Significant reductions in the asphalt layer thickness of the pavement are achieved when wander is considered.

## 1. Introduction

In the modern era, where vehicles are guided by sophisticated navigation systems (known as Autonomous Vehicles) and can communicate with one another (referred to as Connected and Autonomous Vehicles—CAVs), there is a potential to mitigate premature localised road distresses. This can be achieved by leveraging the precise control systems of these vehicles to optimise their tracking on the road, aiming to distribute the wear on the pavement as uniformly as possible. Such practices are expected to significantly enhance pavement life globally.

Many benefits have been attributed to self-driving vehicle technology, including the potential to reduce traffic accidents and fuel consumption and improve the efficiency of road transportation systems [1,2,3]. Due to the fast progress of technology and the implementation of CAVs, a significant concern will shortly be related to the infrastructure required and pavement damage associated with these vehicles’ circulation [4].

The wheel path is the pavement surface area where most vehicle wheel passages are concentrated. In contrast, wheel wander, or the lateral distribution of wheel loads, is a natural phenomenon observed on roadways. The randomness of wheel tracking depends on the vehicle types, individual driving habits, wind effects, and the trailer’s mechanical alignment [5]. Conversely, the circulation of CAVs in roadways will entail changes in the current traffic flow and, consequently, the load positions that pavement structures are subject to [1].

The lateral position is a random phenomenon when trucks are driven by human drivers, considering that they do not follow the same path during the travel. Human-driven trucks follow a normal distribution of lateral positions in the lanes [6]. On the other hand, CAVs tend to circulate continuously at the same distance from the lines that limit the traffic lanes, causing an increase in structural degradation as they constantly circulate in the same transversal road position [7,8,9]. In this way, the frequency with which a point in the pavement is loaded represents a concern in pavement performance. Gungor [10] stated that the lateral position of wheel load or wheel wander must be considered in the pavement design and analysis since its value changes randomly and influences the occurrence of pavement damage over time.

Some studies were conducted to quantify and understand how wheel wander affects pavement premature failure, and the means that the CAV’s adequate wander control can extend pavement life.

Through a finite element model, the CAV lateral distribution effects on fatigue and rutting damage compared to human-driven trucks were evaluated [11]. The simulation included the CAV lateral control modes, such as zero-wander mode (CAVs are centred within the lane like trains), uniform mode, double peak Gaussian mode, and two-section uniform mode. Relative to the human-driven trucks, from the left lane marking, the CAV lateral position is fixed as the centre of the truck’s left dual-wheel at distances of 4.10 cm to 12.70 cm. Zero wander mode condition promoted a highly concentrated distribution of wheel tracks and negatively affected pavement performance, while uniform, double peak Gaussian, and two-section uniform modes contributed positively. For instance, in zero wander mode, the rutting depth will reach 15 mm in just 1.56 years, and fatigue damage will increase by 146%. Also, the two-section uniform mode could reduce fatigue damage by up to 35%.

Chen et al. [12] stated that the CAV’s positions must be adequately programmed to prevent fatigue damage due to CAV circulation. The authors proposed a CAV lateral position adjustment scheme to minimise fatigue damage. Through simulations with the fatigue-oriented method, they concluded that fatigue damage could be reduced by 28%.

In the current traffic flow, the axle spaces and the distance among the trucks promote rest periods of load, allowing the pavement to recover [13]. On the other hand, CAV circulation in a platoon can considerably reduce pavement fatigue life and increase rutting depth occurrence [14].

CAVs in platoons contribute to road safety improvement, and fuel consumption can be reduced by 15%. In contrast, the channelled traffic and load concentration are of concern and can result in accelerated pavement damage. Channelled traffic in zero wander mode could increase fatigue damage by 60% and rutting by 25% [15]. Studies proved that the lateral position of the CAV platoon in the traffic lane can be optimised, thus lessening pavement damage. Okte and Al-Qadi [15] found that, compared with human-driven trucks, the distributed lateral position could decrease fatigue damage by 40% and rutting by 20%.

Fahad and Nagy [16] compared headway distances from 2 m to 5 m truck platoon formation (four trucks per platoon) to estimate the pavement damage effect, considering zero wander and uniform wander modes. In the simulations with the 3D finite element model, they considered platoon 1, which consisted of semi-trailers, platoon 2, which consisted of rigid body trucks, and platoon 3, which consisted of an equally distributed random traffic mix. Results showed that headway distance influenced the pavement strain accumulations. Also, distances less than 3 m caused extreme pavement damage in zero wander mode. However, headway distance had a less significant effect in uniform wander mode. Platoon 3 resulted in minor pavement strain accumulations among the other ones. For this type, the headway distance of 4 m in zero wander mode reduced pavement fatigue damage by eight months. Considering a headway distance of 5 m in zero wander mode and 3 m in uniform wander mode, the fatigue life decreased by 1.2 years and the rutting by 1.6 years, respectively. The study recommended that truck platoon designs consider the classes of trucks typically used on the roadway.

From simulated scenarios of different combinations of CAVs and human-driven trucks for some traffic levels, Merhebi et al. [17] evaluated the asphalt pavement performance relative to fatigue and rutting. The authors concluded that the best scenario for pavement performance was achieved when the quota of CAVs in the traffic flow was 50% using one roadway lane.

Georgouli et al. [18] indicate that zero-wheel wander of AV causes an increased pavement accumulation rate and has a negative structural impact on flexible pavements compared to non-autonomous vehicles. Based on a critical analysis of the literature, it was concluded that wander positively impacts rutting (30–56% reduction). On the other hand, in the case of fatigue cracking, zero wander mode leads to a significant acceleration rate of fatigue damage, even in the range of 2 to 3.

Georgouli and Plati investigated the impact of wheel wander on fatigue and rutting performance of flexible pavements [19]. The researchers showed the negative effects of autonomous tracks with zero wander in the case of fatigue damage and rutting. For all analysed wander distributions, i.e., normal, uniform and Laplace, a reduction in fatigue cracking and rutting damage was observed. The amount of this reduction depends, among other things, on the thickness and stiffness of the asphalt layers. However, in the case of flexible pavements, wheel wander distribution has a more significant influence on rutting. For instance, for every 1000 MPa increase in HMA stiffness, a weighted average decrease of rutting damage reduction by 1.2% for static and 16.3% for dynamic analyses was reported.

In a series of FEM simulations, Yeganeh et al. [20] studied the impact of load distribution on pavement rutting for automated vehicles. The lane width was also considered. Research has shown that the maximum rutting position differs for different loading schemes. In the case of normal wander distribution, maximum rutting occurs at the centre and for zero and uniform distributions at the load edges. Uniform wander distribution of AV traffic in analysed cases could decrease total rutting depth compared to zero wander distribution by 42.35% to 30.54% for the lane width from 3.5 m to 3 m.

The studies confirmed the negative impact of the CAV’s zero lateral wheel wander on pavement damage. Many analyses were developed considering wheel wander distributions (normal, uniform, and others). Pavement life can be extended under certain conditions (optimal wheel wander distribution). However, the impact of the CAV on pavement performance still needs to be fully agreed upon. In this way, other methodologies must be applied to evaluate the ideal lateral wandering pattern for CAVs. Researchers notice potential benefits to pavement life that could result from controlling the lateral position across the road of autonomous trucks and, thanks to this, more uniform usage of the pavement surface [21].

Although some studies have quantified how wheel wander affects pavement premature failure, no model has been developed to express the wandering effect on pavement life as a function of the asphalt layer thickness and subgrade stiffness, the most essential variables that influence the variation of the pavement life due to the traffic wander.

The motivation to undertake the research was the lack of models that would allow for a consideration of wander during pavement design, where the traffic is considered by number of Equivalent Single Axle Loads (ESALs), and which to be used for Connected and Autonomous Vehicles (CAV) to reduce pavement damage and, consequently, extend pavement life.

## 2. Objective

The primary focus of this article is to explore the impact of traffic wander on pavement life to develop a model that estimates how variations in traffic wander, asphalt layer thickness, and subgrade stiffness increase pavement life for cracking and rutting. The traffic wander was considered through the normal distribution and the uniform distribution (recommended by researchers [21,22] as preferred), simulating the human-driven and traffic distribution that minimises the pavement damage. For the model development, influence functions were determined to understand how the traffic wander, the asphalt layer thickness and the subgrade stiffness influence the pavement life. For application in pavement design, the model expresses the variation of the pavement life as a function of the abovementioned parameters.

The developed model was used to create damage influence functions that allow a fast observation of the traffic wander on pavement life variation. The model was also used to calculate the asphalt layer thickness for different traffic levels expressed in terms of Equivalent Single Axle Loads that allow to observe the thickness reduction due to the traffic wander.

## 3. Methodology

### 3.1. Asphalt Pavement Modelling Approach

**Figure 1:**
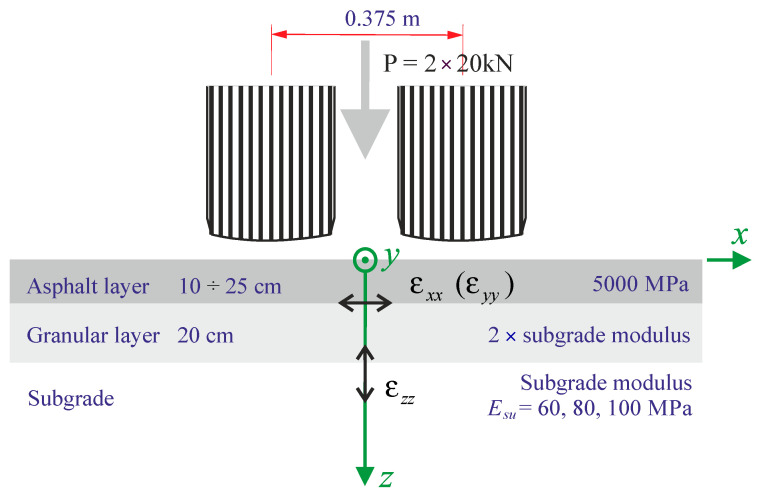
Diagram of the analysed road pavement structures.

Several steps are necessary to determine the fatigue life of a pavement. As it is known, the durability of a road pavement depends mainly on the arrangement of layers constituting the pavement (their mutual arrangement and the thickness of individual layers), the material properties of individual layers (i.e., stiffness), and the properties of the subgrade. For this analysis, it was assumed that the properties of individual road pavement layers are described by constitutive relations of linear elasticity, assuming the isotropy of individual layers. It was assumed that the road surface would be modelled as a system of two layers (asphalt layer and granular layer) resting on an elastic foundation (subgrade with appropriate parameters). The adopted structure scheme and the geometric, stiffness, and loading parameters are shown in Figure 1, varying the asphalt layer thickness ($h_{as}$) for 10, 15, 20, and 25 [cm] and the subgrade stiffness ($E_{su}$) for 60, 80, 100 [MPa]. The stiffness of the asphalt layer was chosen as 5000 MPa (representative of a pavement temperature of 20 °C and 10 Hz), representing a typical value for pavement design in most countries with warm conditions [23,24]. The Poisson’s ratio value was assumed ν=0.35. For the granular layer, a thickness of 20 cm and a stiffness twice that of the subgrade were considered, which are representative of a pavement with one granular layer. Regarding loading, the 80 kN Standard Axle was considered and modelled by two 20 kN loads (single axle with double tyres).

According to the mechanistic-empirical design of structures concerning fatigue cracking, tensile strains at the bottom of the asphalt layer are decisive. As part of the analysis, the transverse strain component was determined, i.e., $\varepsilon_{xx}$ together with longitudinal strain component $\varepsilon_{yy}$, as a function of the spatial variable x, i.e., $\varepsilon_{xx}(x)$ and $\varepsilon_{yy}(x)$. In turn, damage due to rutting is determined by the compressive strain in the direction of the *z*-axis at the top of the pavement subgrade $\varepsilon_{zz}(x)$. For this calculation, software (JPav v. 3.9) developed by one of the co-authors (Jorge Pais) was used, based on the analytical solution of a layered half-space loaded uniformly in a circular area and using the assumption of axial symmetry. The solution obtained in this way is transformed into a Cartesian coordinate system. In the Cartesian system, a translation is then performed relative to the *x*-axis, which allows this solution to be treated as the so-called Green’s influence function. Thanks to this procedure, it is possible to combine loads (e.g., from tandem wheels) using the principle of superposition of stresses, strain and displacements, which applies only to linearly elastic materials and the theory of small deformations (as in the analysed case). Although the program is based on an analytical solution, the result is given in a discrete version (function values in points), which means that all further analysed strain functions are piecewise linear.

### 3.2. Pavement Damage Analysis

Damage due to fatigue cracking was taken into account using Shell’s formula for tensile strains at the bottom of the asphalt layer

(1) $$\varepsilon_{nn}=\left(0.856V_{b}+1.08\right)S_{mix}^{-0.36}N_{n}^{-0.2}$$

where $\varepsilon_{nn}=\varepsilon_{xx}\;or\;\varepsilon_{yy}\;$ is the tensile strains at the bottom of the asphalt layer in xx direction or in the yy direction, $V_{b}$ is binder content, $S_{mix}$ is the norm of the complex modulus of stiffness of the asphalt mixture [Pa], while $N_{n}$ stands for pavement fatigue life due to the horizontal strain $\varepsilon_{nn}$, for the ESAL (Equivalent Single Axle Load). Using the above equation, pavement fatigue lives $N_{x}(x)$ and $N_{y}(x)$ were determined (based on the solution of layered pavement task respectively on $\varepsilon_{xx}(x)$ and $\varepsilon_{yy}(x)$). Additionally, the unit damage functions that are the inverse of the number of cycles to failure are introduced:

(2) $$D_{xx}\left(x\right)=1/N_{x}(x)\;\mathrm{and}\;D_{yy}\left(x\right)=1/N_{y}(x)$$

Similarly, based on the fatigue law for permanent deformation due to the subgrade,

(3) $$\varepsilon_{zz}=a\;N_{z}^{-0.25}$$

The unit damage function was determined due to rutting in the form:

(4) $$D_{zz}\left(x\right)=1/N_{z}(x)$$

where $a=1.8\times 10^{-2}$ assuming a 95% confidence level, while $N_{z}$ means fatigue life due to rutting.

**Figure 2:**
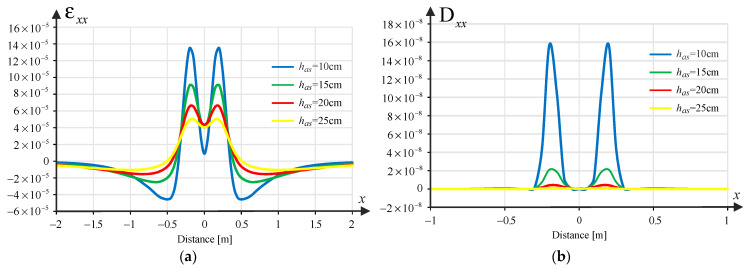
(**a**) Strain component $\varepsilon_{xx}(x)$; (**b**) the resulting unit damage function $D_{xx}\left(x\right)$ depending on the thickness of the asphalt layer.

**Figure 3:**
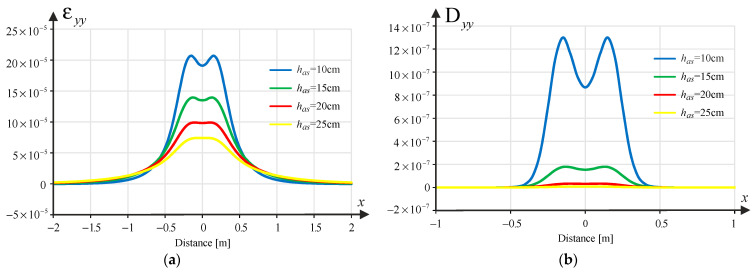
(**a**) Strain component $\varepsilon_{xx}(x)$; (**b**) the resulting unit damage function $D_{yy}\left(x\right)$ depending on the thickness of the asphalt layer.

**Figure 4:**
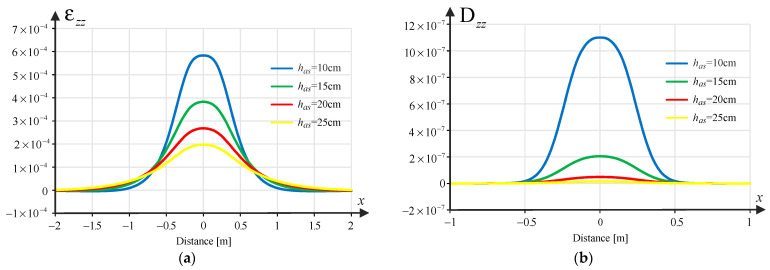
(**a**) Strain component $\varepsilon_{zz}(x)$; (**b**) the resulting unit damage function $D_{zz}\left(x\right)$ depending on the thickness of the asphalt layer.

Figure 2, Figure 3 and Figure 4 show samples of the strains $\varepsilon_{xx}\left(x\right)$, $\varepsilon_{yy}(x)$ and $\varepsilon_{zz}(x)$ and the corresponding damage functions $D_{xx}\left(x\right)$, $D_{yy}\left(x\right)$ and $D_{zz}\left(x\right)$ for the different asphalt layer thicknesses (10, 15, 20 and 25 cm) for the pavement with a subgrade of 80 MPa. Binder content $V_{b}=10\%$ was assumed in the calculations.

### 3.3. Inclusion of Wander Effect

The wander effect was considered by introducing appropriate statistical distribution functions $\tilde{f}\left(x\right)$. Normal distribution (*nd*) and uniform distribution (*ud*) were considered herein. In general, the total impact of the wander effect on pavement damage can be calculated as:

(5) $$D_{nn}^{w,dt}(x)=\int_{-\infty }^{\infty }\tilde{f}\left(x\right)D_{nn}\left(x-\eta \right)d\eta$$

where $D_{nn}^{w,dt}(x)$ is the pavement damage due to the wander for the distribution type dt (*nd* for normal distribution and *ud* for uniform distribution) and due to the strain nn (xx, yy and zz), and the parameter η determines the location of $D_{nn}\left(x\right)$ relative to the global coordinate system. As the results regarding the strain components and, consequently, the damage parameters $D_{nn}$ are determined in the discrete version, the above integral was calculated numerically using the Excel package. As the integral functions converge to zero relatively quickly, the integration limits were changed from infinity to finite values, i.e., upper and lower integration limits were adopted, respectively $S_{p}=\pm 2.60\left[m\right]$. This limit corresponds to 2.00 m, which is the maximum influence distance of a load in a pavement plus the maximum wander considered in this work (0.60 m).

The calculations were performed by determining the function $D_{nn}^{k}\left(x-\eta \right)$ in 105 points ($k\in \left[1,\;105\right]$), from x=−2.60 m up to 2.60 m with an increment of 0.05 m, and function $\tilde{f}\left(x_{i}\right)$ in 25 points ($i\in \left[1,\;25\right]$) corresponding to the wander steps from −0.60 m up to 0.60 m, with an interval of 0.05 m. The interval 0.05 m is small enough to obtain a complete discretization of the function. This allowed building matrix $A_{i,k}$ which elements can be determined from the following formula:

(6) $$A_{i,k}=\tilde{f}\left(x_{i}\right)D_{nn}^{k}\left(x_{k}\right)$$

Total damage considering the wander effect for a given location $x_{k}$ is determined by summing the elements in each column of matrix $A_{i,k}$ (Equation (6)) for the 25 points considered in the analysis:

(7) $$D_{nn}^{w,dt}\left(x_{k}\right)=\sum_{i=1}^{N}\tilde{f}\left(x_{i}\right)D_{nn}^{k=i}\left(x_{k}\right)$$

**Figure 5:**
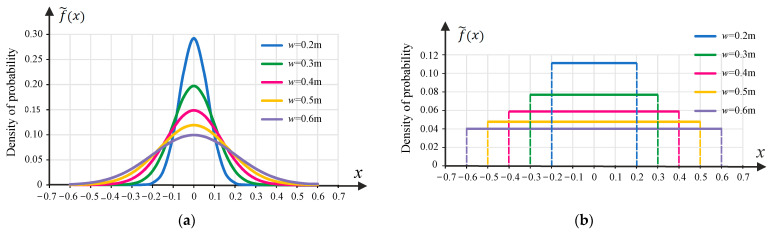
Probability density functions in the case of: (**a**) normal distribution, (**b**) uniform distribution.

In Equations (6) and (7), $\tilde{f}\left(x_{i}\right)$ is the probability density function. The probability density functions used (normal distribution and uniform distribution) are shown in Figure 5 for a wander of 0.20 m up to 0.60 m.

Finally, the relative pavement damage can be calculated by dividing the damage due to the wander by the damage without wander.

As the main objective of is the research was to establish a model to define the relative life of the pavement as a function of the wander, it is necessary in this case to have the inverse of the damage of the pavement, that is, the relative life of the pavement is given by the damage when there is no wander divided by the damage when there is wander, as expressed in Equation (8):

(8) $$F_{nn}^{w,dt}=\frac{max(D_{nn}^{w=0}(x))}{max(D_{nn}^{w,dt}(x))}$$

Note that the parameter obtained from the above equation is a scalar due to the maximum function appearing in the numerator and denominator. Function $D_{nn}^{w=0,dt}(x)$, in the numerator of Equation (8), is the pavement damage when the wandering effect is not considered.

**Figure 6:**
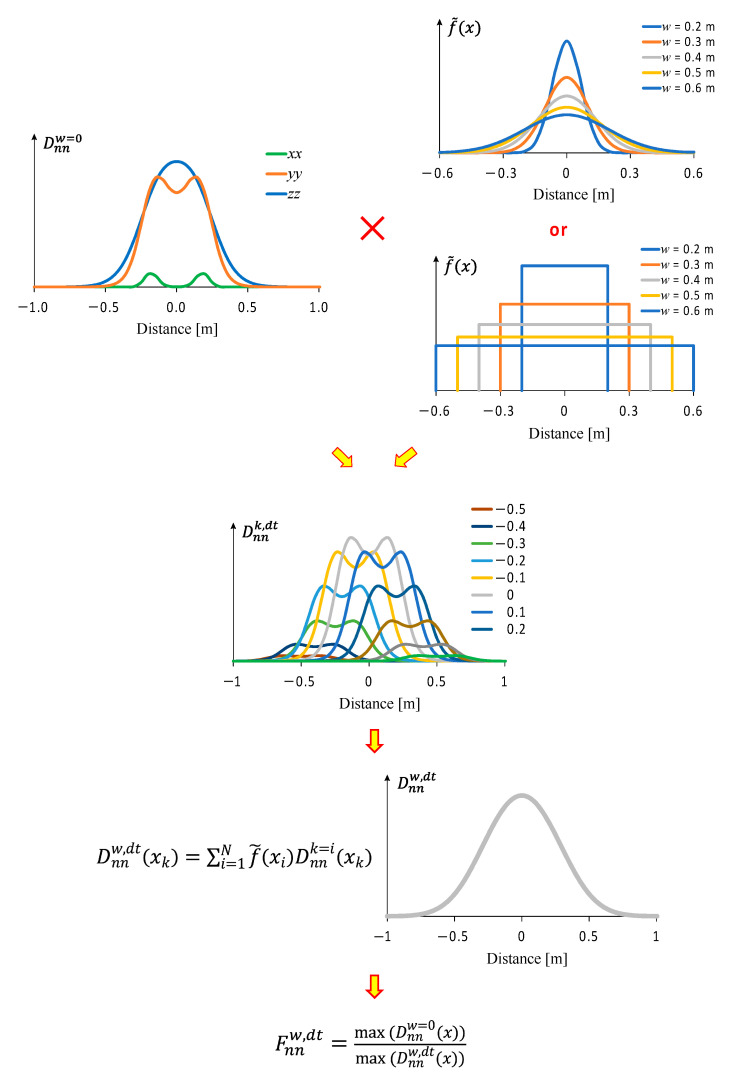
The concept of performing numerical calculations to find the result of the integral (5) needed to determine the relative pavement life, taking into account the wandering effect.

The concept of the numerical calculations (numerical integration) using Excel, Equations (6) and (7) and probability density distributions to calculate the relative pavement life (pavement life considering traffic wander compared to pavement life without traffic wander) presented in Figure 5 is illustrated in Figure 6.

Using the method presented above, the pavement life influence function values variation was determined for a wander (w) of 0, 0.2, 0.3, 0.4, 0.5, and 0.6 [m]. A set of 60 solutions was obtained corresponding to the factorial of the pavement configuration (changing the asphalt layer thickness and the subgrade) for these five wander configurations.

**Figure 7:**
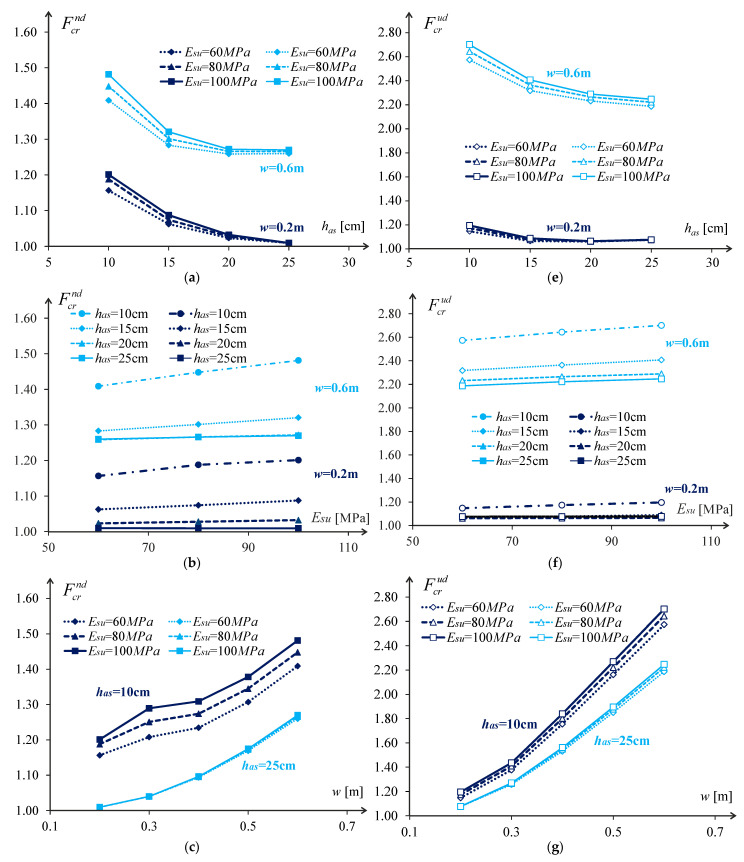

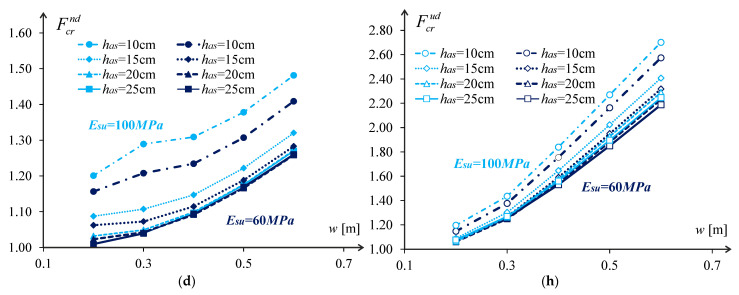
Relative pavement life influence functions for cracking: (**a**–**d**) for dt=nd; and (**e**–**h**) for dt=ud.

**Figure 8:**
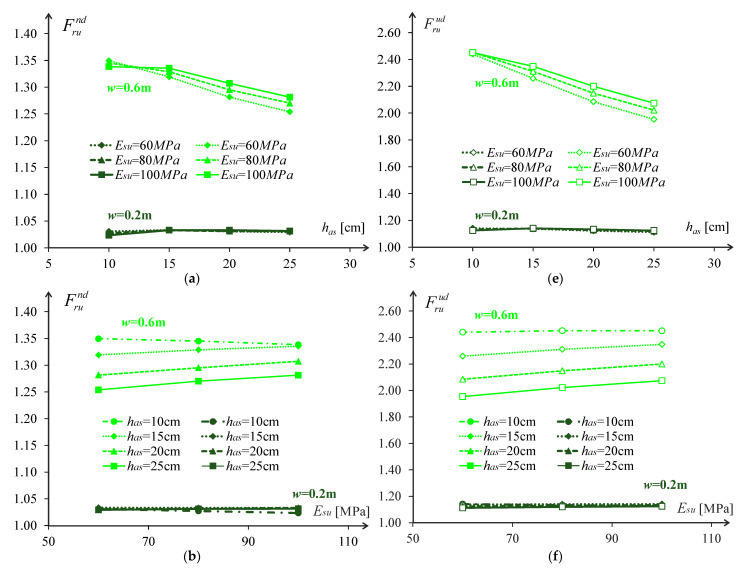

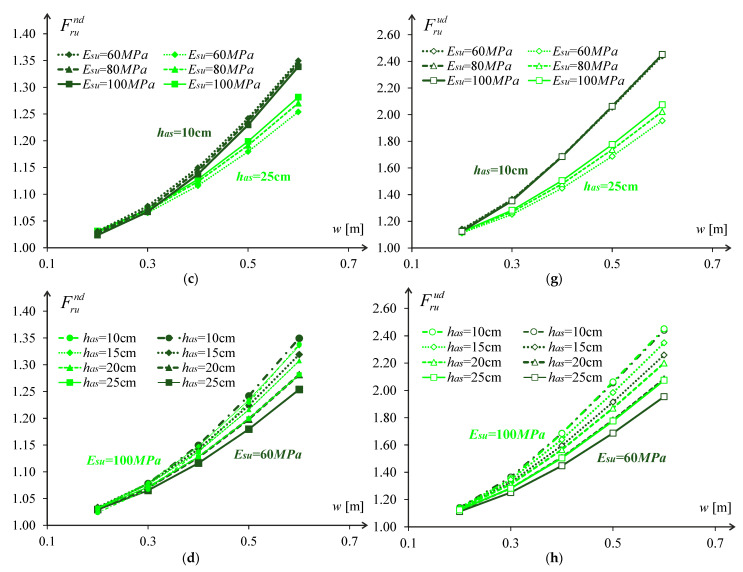
Relative pavement life influence functions for rutting: (**a**–**d**) for dt=nd; and (**e**–**h**) for dt=ud.

Examples of the relative pavement life are plotted in Figure 7 and Figure 8 as a function of the wander and asphalt layer thickness for different pavement configurations. These plots define the typical influence of the studied parameters on the pavement’s relative life. This typical influence in the case of the analysed pavements is characterised by:

1. The increase of the asphalt layer thickness reduces the relative pavement life following a second-degree polynomial law;
2. The rise in the subgrade stiffness increases the relative pavement life following a linear law;
3. The increase of the wander increases the relative pavement life following a second-degree polynomial law.

So, developing a model to define the relative pavement life due to the variation of the pavement constitution and the wander will be based on these assumptions.

## 4. Relative Pavement Life Influence Functions

Based on the calculations presented earlier, the influence of the wander, thickness of the asphalt layer, and stiffness of the subgrade, on the relative pavement life for fatigue cracking and rutting were obtained (Table S1). This influence was defined for discrete values of these parameters, so it is essential to determine laws that define this influence continuously, allowing them to be applied to any value of these parameters.

One option for developing these models is Artificial Neural Networks (ANN), which can adjust any variable to the most varied set of input data. Artificial neural models were used for the data obtained in this study, showing excellent agreement between the ANN prediction results and the input data results [25]. Unfortunately, this model (ANN) is the so-called black box, where it is impossible to understand the effect of one variable on the final result. For example, as observed in previous figures, the increase in the asphalt layer thickness reduces the relative pavement life following a second-degree polynomial law. This effect in a mechanistic model is expressed by a second-degree polynomial law, but in ANN is only expressed by a complex set of additions and multiplications.

Therefore, the main objective of the research was to establish a model for the relative life of pavements due to wander that expresses the influence of each variable considered in the model, in this case through polynomials (second-degree) equations.

As shown in [26], quadratic and linear functions can approximate the relationship between the parameters characterising failure due to fatigue cracking and rutting. However, using a quadratic function to consider the influence of subgrade stiffness on pavement damage allows for the improvement of model quality.

Relative pavement life functions were determined depending on the wander parameter w, asphalt layer thickness $h_{as}$ and stiffness of the subgrade $E_{su}$. Therefore, the form of the approximation function was proposed as a product of quadratic functions depending on individual variables as the following:

(9) $$F_{nn}\left(w,h_{as\;},E_{su}\right)=(a_{1}+b_{1}w+c_{1}w^{2})(a_{2}+b_{2}h_{as}+c_{2}h_{as}^{2})(a_{3}+b_{3}E_{su}+c_{3}E_{su}^{2})$$

The proposed function, defined as the product of quadratic functions with respect to individual variables, was developed following a prior analysis of the determination coefficients for linear, quadratic, and cubic functions with respect to the variables w, $h_{as}$, and $E_{su}$ (assuming the constancy of the other two variables). It was found that, in some cases, a reasonable fit is achieved even with a linear function. However, adopting a quadratic function significantly improved the R^2^ determination coefficient, while the use of a cubic polynomial resulted in only a marginal increase in R^2^. Consequently, the decision was made to adopt an approximating function as the product of quadratic functions, with the assumption that, in cases where a linear function is sufficient, the coefficients of the quadratic terms would take very small values, for the approximation with respect to $E_{su}$. While approximations using non-polynomial functions could potentially be explored, this issue was not addressed in the present study.It is worth noting that the parameters of the above approximation function will be sought in four cases, i.e., for the relative pavement life function characterizing fatigue cracking $F_{cr}\left(w,h_{as},E_{su}\right)$ and relative pavement life function due to rutting $F_{ru}\left(w,h_{as},E_{su}\right)$ also taking into account two statistical distributions regarding the wandering effect (normal (*nd*) and uniform (*ud*)), leading to the following designations were introduced: $F_{cr}^{nd}\left(w,h_{as},E_{su}\right)$, $F_{cr}^{rd}\left(w,h_{as},E_{su}\right)$, $F_{ru}^{nd}\left(w,h_{as},E_{su}\right)$ and $F_{ru}^{rd}\left(w,h_{as},E_{su}\right)$.

The Wolfram Mathematica analytical and numerical calculation system and the NonlinearModelFit with the Levenberg–Marquardt variant of the Gauss–Newton method were used to determine the parameters in the approximation functions.

As observed in Figure 7 and Figure 8, each of the variables considered in this study leads to an increase in the relative pavement life, so each individual function ($F_{nn}$) must lead to a value greater than one. This was ensured considering the wander, the thickness of the asphalt layer, and the stiffness of the subgrade through their relative values to the maximum value considered in the study. Thus, these three variables were considered as:

(10) $$\tilde{w}=\frac{w}{w^{max}}=\frac{w}{0.60\;m}$$

(11) $${\tilde{h}}_{as}=\frac{h_{as}}{h_{as}^{max}}=\frac{h_{as}}{0.25\;m}$$

(12) $${\tilde{E}}_{su}=\frac{E_{su}}{E_{su}^{max}}=\frac{E_{su}}{100\;\mathrm{MPa}}$$

Thus, the model proposed in Equation (9) is expressed as:

(13) $$F_{nn}\left(w,h_{as},E_{su}\right)=({\tilde{a}}_{1}+{\tilde{b}}_{1}\tilde{w}+{\tilde{c}}_{1}{\tilde{w}}^{2})({\tilde{a}}_{2}+{\tilde{b}}_{2}{\tilde{h}}_{as}+{\tilde{c}}_{2}{\tilde{h}}_{as}^{2})({\tilde{a}}_{3}+{\tilde{b}}_{3}{\tilde{E}}_{su}+{\tilde{c}}_{3}{\tilde{E}}_{su}^{2})$$

The parameters ${\tilde{a}}_{i}$, ${\tilde{b}}_{i},$ and ${\tilde{c}}_{i}$ of Equation (13) were obtained as indicated in Table 1.

**Figure 9:**
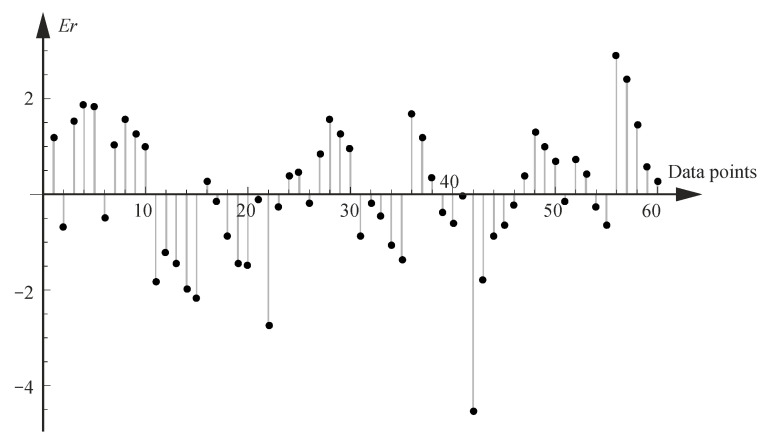
Relative error in % according to Equation (14) for function $F_{cr}^{nd}$.

Two additional tests were proposed to evaluate the obtained results. In the first case, the error in mapping the discrete result constitutes the basis for the approximation by the approximation function, i.e., calculated by Equation (14) for the cracking considering the normal distribution. As observed in Figure 9, for this case, the error between the discrete values used to develop the model and those predicted by the model is less than 2%. For the other models, the error is also within 2%.

(14) $$Er=\frac{F_{cr}^{nd}-F_{cr,discret}^{nd}}{F_{cr,discret}^{nd}}100\%$$

**Figure 10:**
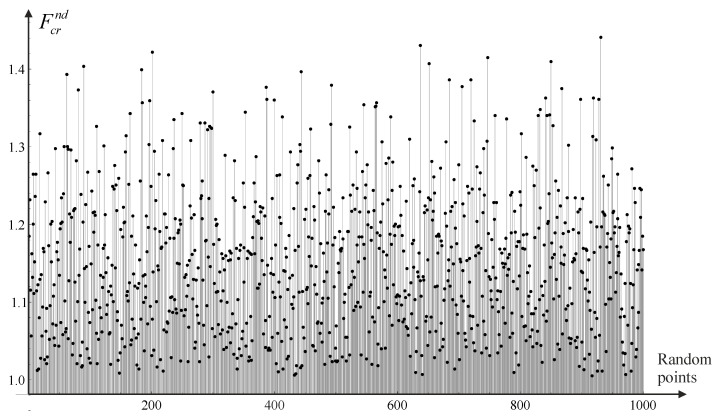
Values of the function $F_{cr}^{nd}$ for 1000 sets of random data from a range of approximation.

The second test aims to check whether the values predicted by the approximation functions are within the range determined by the data used for approximation. This test is intended to verify whether we are dealing with the so-called phenomenon of overfitting, which, in the case under consideration, may appear to be especially concerning a variable $E_{su}$ (only three data points). For this purpose, a procedure was programmed to generate values of approximation functions for a set of 1000 data randomly selected from the range of variables, i.e., (w∈[0.2, 0.6][m], $h_{as\;}\in \left[0.10,\;0.25\right][m]$, $E_{su}\in \left[60,\;100\right][MPa]$). For example, Figure 10 shows the results obtained in the case of function $F_{cr}^{nd}$ which values according to Table 1 should be in the range [1.01, 1.48]. Analysis of the data presented in Figure 10 confirms that the function values for randomly generated data are within the indicated range. The same type of test was performed for all four functions, confirming the correctness of the proposed approximations.

## 5. Results Analysis and Discussion

**Figure 11:**
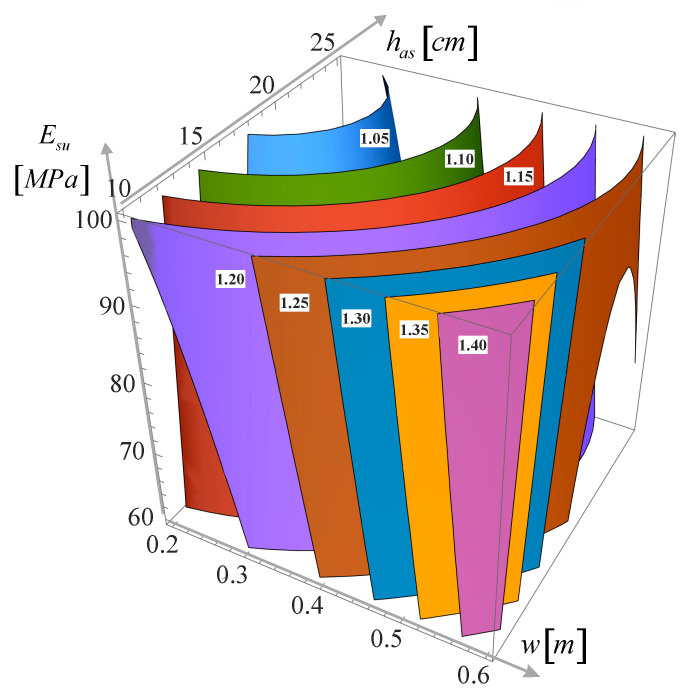
Contour graph of $F_{cr}^{nd}\left(w,h_{as},E_{su}\right)$.

This chapter presents the influence of the three variables considered in this study on the relative pavement life considering the cracking for the normal distribution of the wander $F_{cr}^{nd}\left(w,h_{as},E_{su}\right)$. In the analysis shown above, the influence was expressed only by varying one variable at a time. However, it is interesting to see how the relative pavement life varies depending on the combination of the three variables used in the model (wander, asphalt layer thickness and subgrade stiffness). This is presented in Figure 11 in a 3D graph in which each axis represents each of the variables studied, and the graph presents contours for relative pavement life variation values from 1.05 to 1.40. In this graph, the second-degree polynomial variation of the relative pavement life with the wander and the asphalt layer thickness is visible, while the subgrade stiffness has a linear influence on the relative pavement life (even though the approximation was made with a second-degree polynomial).

**Figure 12:**
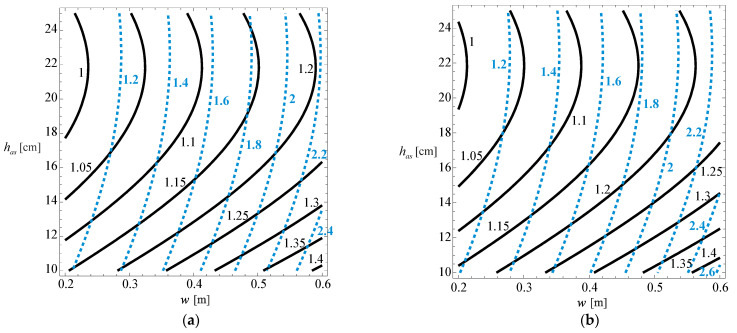

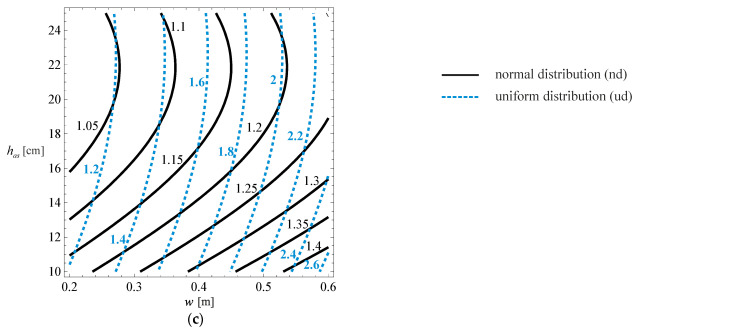
Relative pavement life influence functions $F_{cr}^{nd}\left(w,h_{as},E_{su}\right)$ and $F_{cr}^{rd}\left(w,h_{as},E_{su}\right)$ for (**a**) $E_{su}$= 60 [MPa], (**b**) $E_{su}$= 80 [MPa] and (**c**) $E_{su}$= 100 [MPa].

**Figure 13:**
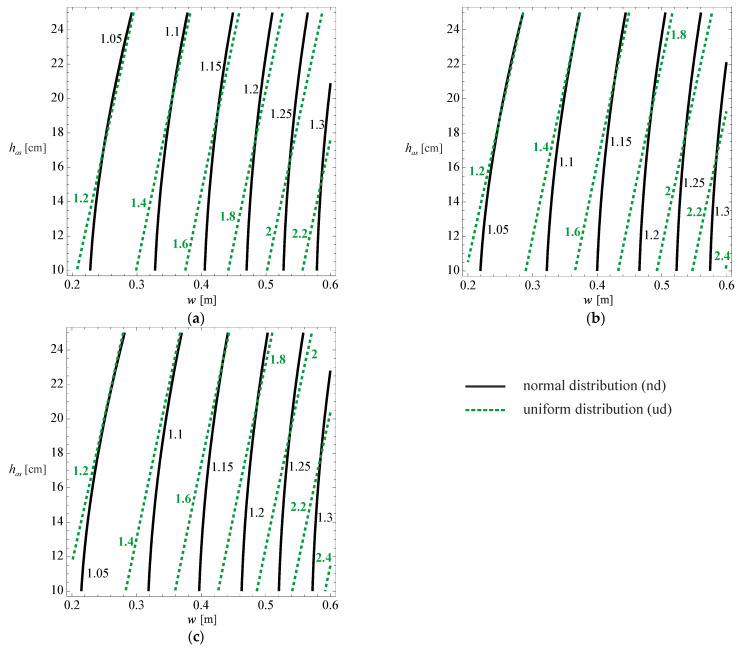
Relative pavement life influence functions $F_{ru}^{nd}\left(w,h_{as},E_{su}\right)$ and $F_{ru}^{rd}\left(w,h_{as},E_{su}\right)$ for (**a**) $E_{su}$= 60 [MPa], (**b**) $E_{su}$= 80 [MPa] and (**c**) $E_{su}$= 100 [MPa].

Despite the importance of the 3D graph, for practical application (for those who do not intend to use the developed model, or want to understand how the various variables influence the developed model), 2D contours are presented that relate the relative pavement life with the asphalt layer thickness and the wander, for the multiple cases considered in the developed model (cracking for normal distribution, cracking for uniform distribution, rutting for normal distribution and rutting for uniform distribution). These 2D graphs are presented in Figure 12 and Figure 13 for the three subgrade stiffness values considered in the development of the model (60, 80, and 100 MPa), typical values found for subgrade stiffness in road pavements.

In these figures, the continuous line represents the normal distribution, while the interrupted line represents the uniform distribution.

By analysing Figure 12 for a wander of 0.60 m, it is possible to verify that the relative cracking life of the pavement, considering a normal wander distribution, can reach 1.4 times the life if there was no wander. For wanders of the order of 0.10 m, the relative life of the pavement is almost unchanged compared to the condition with no wander.

For the uniform distribution, there is a significant increase in the pavement life due to wander, and the life can be doubled for high values of wander.

Regarding pavement life due to rutting (Figure 13), it is evident that the impact of wander and asphalt layer thickness on pavement life variation is non-linear but may be easily approximated with a linear function without a big error. Such a trend is very similar to that observed for cracking.

## 6. The Impact of Considering Vehicle Control on Pavement Life

The variation in relative pavement life considering the wander effect produced by normal and uniform distributions were studied. These distributions can be considered the most frequent (normal distribution) and the one that would cause the least damage to the pavement (uniform distribution).

Furthermore, the normal distribution can be considered the one that current users adopt, while the uniform distribution can be considered the one that optimises the behaviour of the pavements through the total control of driving. Therefore, it is important to understand the benefits of total control of vehicles to minimise damage to pavements and consequently increase the pavement life.

To evaluate the effect of vehicle control, the relative increase of pavement life, ${Spr}_{nn}$, indicated in Equation (15) was defined in which this effect is calculated by the difference in the relative life of the pavement for the uniform distribution compared to the normal distribution

(15) $${Spr}_{nn}=\frac{F_{nn}^{ud}-F_{nn}^{nd}}{F_{nn}^{nd}}100\%$$

where nn=cr (cracking) or ru (rutting).

**Figure 14:**
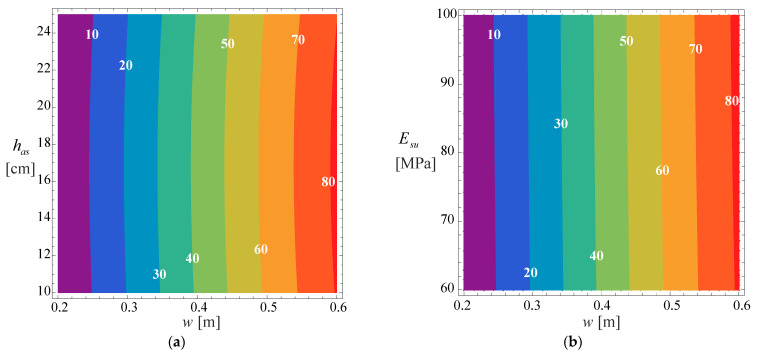
Relative increase in pavement rutting life ${Spr}_{cr}$ (in %) due to the implementation of uniform distribution of the wander in case of: (**a**) $E_{su}=$ 80 MPa, (**b**) $h_{as}=$15 cm.

**Figure 15:**
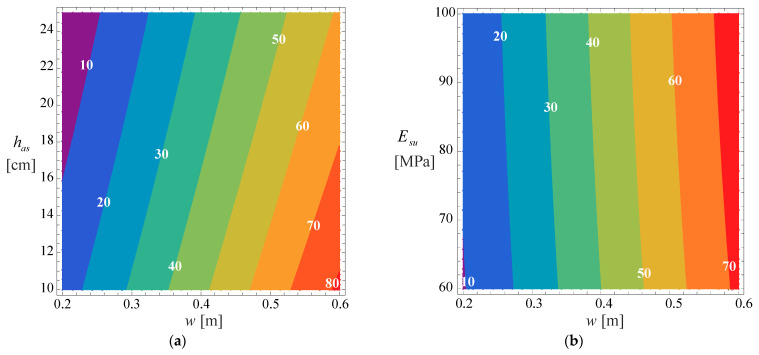
Relative increase in pavement rutting life ${Spr}_{ru}$ (in %) due to the implementation of uniform distribution of the wander in case of: (**a**) $E_{su}=$ 80 MPa, (**b**) $h_{as}=$15 cm.

Figure 14 and Figure 15 show the effect of vehicle control for two cases due to cracking and rutting, respectively. In both cases, relative increases in pavement life are shown at a fixed subgrade modulus $E_{su}=$ 80 MPa (a) in Figure 14 and Figure 15, and with a fixed asphalt layer thickness $h_{as}=$15 cm (b).

These figures allow to conclude that using a normal distribution for the wander allows an increase of 10% in pavement life of wanders of around 0.25 m and for wanders around 0.30 m, the increase in pavement life can be about 20%. Despite the small magnitude of these increments, they significantly affect the pavement because they allow the pavement life to be increased from 20 years to 22 or 24 years, with the inherent consequences of cost savings in the rehabilitation or pavement reinforcement.

## 7. The Impact of Increase Relative Pavement Life on Pavement Structure

This study evaluated the effect of vehicle wander on the relative pavement life compared to conditions where there is no wander. The relative pavement life was calculated for different pavements by varying the asphalt layer thickness and subgrade stiffness. Based on the results obtained, it can be concluded that as the asphalt layer thickness increases, the relative pavement life decreases. This indicates that for pavements with thicker asphalt layers, the effect of wander on pavement life becomes less pronounced. Conversely, an increase in subgrade stiffness leads to an increase in relative pavement life.

As for the wander, its increase leads to an increase in the relative pavement life. Overall, there is an increase in the relative pavement life when a wander is applied to road traffic, which can be 1.5 times for normal distribution and up to 2.7 times for uniform distribution, considering the values used in the simulations of this work. This means that a pavement in these conditions would support 1.5 times or 2.7 times more traffic compared to a situation in which there would be no wander.

**Figure 16:**
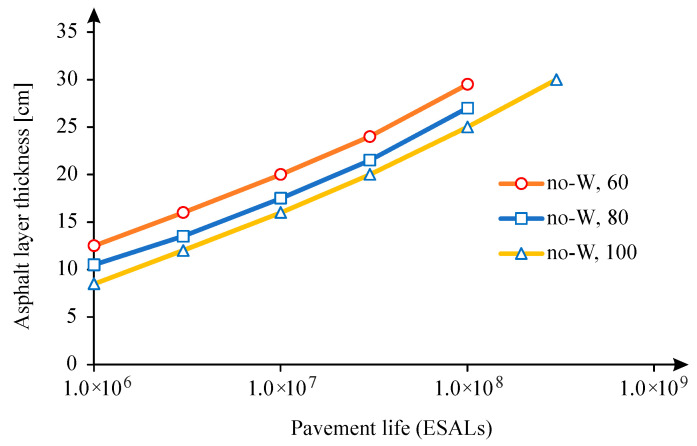
Asphalt layer thickness as a function of the traffic for the no-wander condition (for subgrade modulus 60, 80, and 100 MPa).

It is now important to understand the implications of this increase of the relative pavement life on the pavement structure, mainly in the pavement thickness. For this purpose, the pavement defined in Figure 1 was considered, for which, using the Shell pavement design method, the thickness of the asphalt layer was calculated for six traffic levels expressed in terms of Equivalent Single Axle Load (ESALs), considering 1 × 10^6^, 3 × 10^6^, 10 × 10^6^, 30 × 10^6^, 100 × 10^6^, 300 × 10^6^. The thickness of the asphalt layer was calculated for the three types of subgrade (60, 80, and 100 MPa). The calculation of the thickness of the asphalt layer was limited to 30 cm, and the values indicated in Figure 16 were obtained, verifying that the thickness of the asphalt layer increases almost linearly with the logarithm of traffic. By increasing the stiffness of the subgrade, the thickness of the asphalt layer is reduced. In this figure, caption no-W means that it corresponds to the no-wander situation, while the number corresponds to the subgrade stiffness.

The implications of the relative increase in pavement life were carried out for the two distributions considered for the development of the model (normal and uniform) and for a wander of 0.50 m, which can be considered the maximum possible value to apply in road traffic.

Thus, the pavement thickness (asphalt layer) was calculated for a wander of 0.50 m and for the various traffic levels and subgrade classes, considering both the fatigue cracking of the asphalt layers and the rutting due to the pavement foundation as distress mode.

**Figure 17:**
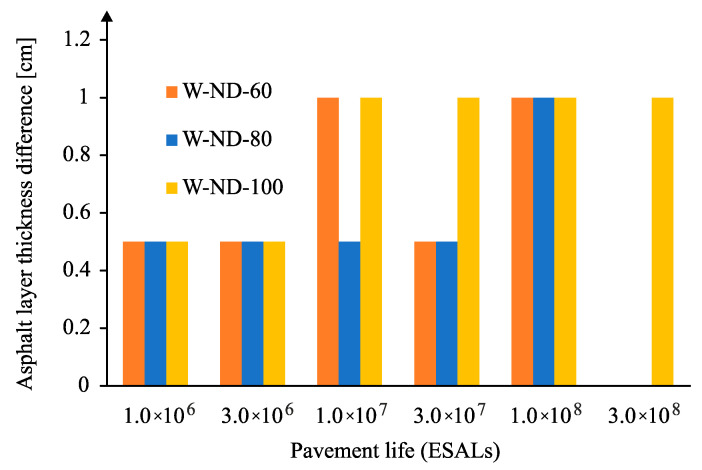
Reduction of asphalt layer thickness for normal wander distribution and rutting (for subgrade modulus 60, 80, and 100 MPa).

In the case of traffic following a normal distribution, the difference in pavement thickness was calculated for the case of rutting (decisive criterion in this case), obtaining the difference in asphalt thickness shown in Figure 17, verifying that it is possible to reduce up to 1 cm in the asphalt thickness, considering this type of lateral traffic distribution. It is also noted that as the traffic level increases, consideration of wander allows the thickness of the pavement to be reduced.

**Figure 18:**
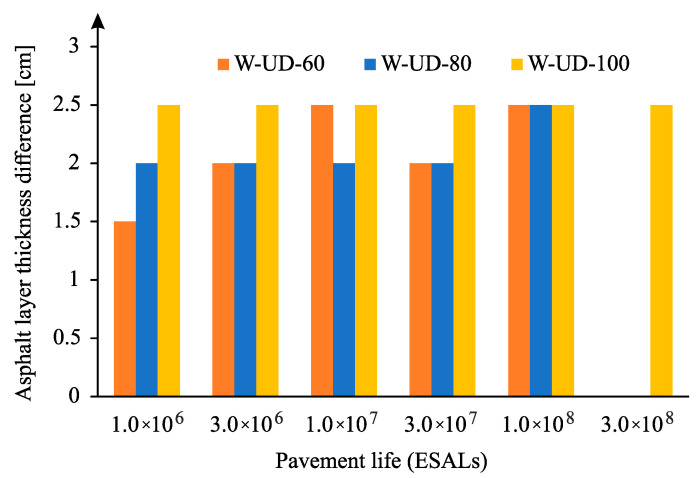
Reduction of asphalt layer thickness for uniform wander distribution and rutting (for subgrade modulus 60, 80, and 100 MPa).

An identical analysis was carried out for the case of uniform traffic distribution and for the case of rutting leading to the asphalt layer reduction indicated in Figure 18, verifying that the decrease in the thickness of the asphalt layer is much more significant than in the case of normal distribution. In this simulation, it reaches 2.5 cm. This fact results from the relative pavement life that, in this case, presents a high value, which reached values greater than 2 when comparing the pavement life with and without wander.

## 8. Conclusions

The research investigated the impact of traffic wander on pavement life, to be used for Connected and Autonomous Vehicles (CAV) to reduce pavement damage and, consequently, extend pavement life. Relative pavement life influence functions were proposed to assess the impact of traffic wander quantitatively. The pavement constitution (thickness of the asphalt layer and stiffness of the subgrade) was also considered in the relative pavement life analysis.

This study emphasises the adjustment of the traffic wander distribution across the transverse direction of the lane. Two specific wander distributions were analysed: normal and uniform. The normal distribution typically reflects human driving patterns, while the uniform distribution is designed to minimise pavement damage and is recommended for use with CAV systems.

The research involved pavement analysis under various configurations, changing the thickness of the asphalt layer and the stiffness of the subgrade. The findings revealed that the relative pavement life (pavement life considering the traffic wander compared to the pavement life without traffic wander) for cracking and rutting exhibits a second-degree polynomial relationship with wander and asphalt layer thickness. An increase in the asphalt layer thickness reduces the relative pavement life, while an increase in wander increases the relative pavement life. In contrast, changes in subgrade stiffness impact pavement life almost linearly. In this case, the increase in the stiffness has a positive impact on the relative pavement life.

These observed influences allowed the development of a model that predicts how variations in traffic wander, asphalt layer thickness, and subgrade stiffness affect the relative pavement life for cracking and rutting for normal and uniform wander distributions. The findings indicated that wander impacts are minimal at approximately 0.10 m but become significantly more pronounced beyond 0.20 m. Proposed relative pavement life influence functions can be easily implemented for the calculation of pavement service life extension using the concept of CAV and may be useful in determining the optimal wander distribution.

The models developed aim to define the variation of the pavement life when traffic wander is applied, that is, how much the pavement life varies when traffic wander is applied. These models, developed to predict both cracking and permanent deformation due to the pavement characteristics, allow:

1. Evaluate the pavement life gain as a function of traffic wander;
2. Define the wander to be applied to autonomous and connected vehicles to maximise pavement life.

The advantage of the presented models is their simplicity. The models can be successfully used not only by researchers but also by designers. Future studies can be carried out to consider a wide range of pavement structures and the loading characteristics of heavy vehicles, as well as different types of wander distributions.

Using the data related to the relative pavement life, an analysis of the pavement constitution for different traffic levels showed the reduction in the asphalt layer thickness that can be obtained when traffic wander is considered, mainly considering its uniform distribution.

## Figures and Tables

**Table 1:** Parameters of approximation functions (Equation (13)).

Variable	Parameter	Unit	Normal Distribution		Uniform Distribution	
		[-]	$F_{cr}^{nd}$	$F_{ru}^{nd}$	$F_{cr}^{ud}$	$F_{ru}^{ud}$
$\tilde{w}$	${\tilde{a}}_{1}$	[-]	1.0130	0.9112	0.6956	0.7866
	${\tilde{b}}_{1}$	[-]	−0.1649	0.2117	0.6180	0.4390
	${\tilde{c}}_{1}$	[-]	0.3777	0.1728	0.8856	0.8181
${\tilde{h}}_{as}$	${\tilde{a}}_{2}$	[-]	1.5701	1.0027	1.5346	1.2728
	${\tilde{b}}_{2}$	[-]	−1.3135	0.00793	−1.2006	−0.2920
	${\tilde{c}}_{2}$	[-]	0.7511	−0.0106	0.6660	0.0192
${\tilde{E}}_{su}$	${\tilde{a}}_{3}$	[-]	0.9525	0.9663	0.9480	0.9218
	${\tilde{b}}_{3}$	[-]	0.0998	0.0680	0.1197	0.1674
	${\tilde{c}}_{3}$	[-]	−0.0215	−0.0196	−0.0237	−0.0617
$R^{2}$		[-]	0.9777	0.9161	0.9990	0.9997
Min (Er)		[%]	−4.54	−3.59	−5.95	−7.94
Max (Er)		[%]	2.90	3.33	4.55	8.47
Min ($F_{nn,discrete}^{nn}$)		[-]	1.005	1.001	1.011	1.024
Max ($F_{nn,discrete}^{nn}$)		[-]	1.472	1.320	2.665	2.434

## Data Availability

The original contributions presented in this study are included in the article/Supplementary Materials. Further inquiries can be directed to the corresponding authors.

## Supplementary Materials

The following supporting information can be downloaded at: https://www.mdpi.com/article/10.3390/ma18112609/s1, Table S1: Relative pavement life for cracking and rutting.

## References

[1] Abdulsattar H., Mostafizi A., Siam M.R.K., Wang H. (2020). Measuring the Impacts of Connected Vehicles on Travel Time Reliability in a Work Zone Environment: An Agent-Based Approach. J. Intell. Transp. Syst. Technol. Planning Oper..

[2] Shiwakoti N., Stasinopoulos P., Fedele F. (2020). Investigating the State of Connected and Autonomous Vehicles: A Literature Review. Transp. Res. Procedia.

[3] Amini E., Omidvar A., Elefteriadou L. (2021). Optimizing Operations at Freeway Weaves with Connected and Automated Vehicles. Transp. Res. Part C Emerg. Technol..

[4] Manivasakan H., Kalra R., O’Hern S., Fang Y., Xi Y., Zheng N. (2021). Infrastructure Requirement for Autonomous Vehicle Integration for Future Urban and Suburban Roads—Current Practice and a Case Study of Melbourne, Australia. Transp. Res. Part A Policy Pract..

[5] Buiter R., Cortenraad W.M.H., van Eck A.C., van Rij H. (1989). Effects of Transverse Distribution of Heavy Vehicles on Thickness Design of Full-Depth Asphalt Pavements. Transp. Res. Rec..

[6] Gungor O.E., Al-Qadi I.L. (2022). Wander 2D: A Flexible Pavement Design Framework for Autonomous and Connected Trucks. Int. J. Pavement Eng..

[7] Erlingsson S., Safwat S., Mcgarvey T. Influence of Heavy Traffic Lateral Wander on Pavement Deterioration. Proceedings of the 4th European Pavement and Asset Management Conference.

[8] Shankar P.R., Lee C.E. (1985). Lateral Placement of Truck Wheels Within Highway Lanes. Transp. Res. Rec..

[9] Blab R., Litzka J. Measurements of the Lateral Distribution of Heavy Vehicles and Its Effects on the Design of Road Pavements. Proceedings of the International Symposium on Heavy Vehicle Weights and Dimensions, Road Transport Technology.

[10] Gungor O.E. (2018). A Literature Review on Wheel Wander.

[11] Chen F., Song M., Ma X., Zhu X. (2019). Assess the Impacts of Different Autonomous Trucks’ Lateral Control Modes on Asphalt Pavement Performance. Transp. Res. Part C Emerg. Technol..

[12] Chen F., Song M., Ma X. (2020). A Lateral Control Scheme of Autonomous Vehicles Considering Pavement Sustainability. J. Clean. Prod..

[13] Pérez-Jiménez F.E., Miró R., Botella R., López-Montero T., Martínez A.H. (2022). The Effect of Temperature, Rest Periods and Ageing on the Response of Bituminous Materials in Fatigue Tests: Considerations and Proposals on Analytical Dimensioning Models. Materials.

[14] Al-Qadi I.L., Okte E., Ramakrishnan A., Zhou Q., Sayeh W. (2021). Truck Platooning on Flexible Pavements in Illinois.

[15] Okte E., Al-Qadi I.L. (2022). Impact of Autonomous and Human-Driven Trucks on Flexible Pavement Design. Transp. Res. Rec..

[16] Fahad M., Nagy R. (2023). Truck Platoon Analysis for Autonomous Trucks. SN Appl. Sci..

[17] Merhebi G.H., Joumblat R., Elkordi A. (2023). Assessment of the Effect of Different Loading Combinations Due to Truck Platooning and Autonomous Vehicles on the Performance of Asphalt Pavement. Sustainability.

[18] Georgouli K., Plati C., Loizos A. (2021). Autonomous Vehicles Wheel Wander: Structural Impact on Flexible Pavements. J. Traffic Transp. Eng..

[19] Georgouli K., Plati C. (2022). Autonomous Trucks’ (ATs) Lateral Distribution and Asphalt Pavement Performance. Int. J. Pavement Eng..

[20] Yeganeh A., Vandoren B., Pirdavani A. (2022). Impacts of Load Distribution and Lane Width on Pavement Rutting Performance for Automated Vehicles. Int. J. Pavement Eng..

[21] Noorvand H., Karnati G., Underwood B.S. (2017). Autonomous Vehicles: Assessment of the Implications of Truck Positioning on Flexible Pavement Performance and Design. Transp. Res. Rec..

[22] Zhou F., Sheng H., Xue W., Flintsch G., Optimizing the Lateral Wandering of Automated Vehicles to Improve Roadway Safety and Pavement Life Report 02-008; 2019, Safe-D National UTC and Texas A&M Transportation Institute. https://safed.vtti.vt.edu/wp-content/uploads/2020/08/02-008_Final-Research-Report_Final.pdf.

[23] Amorim S.I.R., Pais J.C., Vale A.C., Minhoto M.J.C. (2015). A Model for Equivalent Axle Load Factors. Int. J. Pavement Eng..

[24] Pais J., Santos C., Pereira P., Kaloush K. (2020). The Adjustment of Pavement Deflections Due to Temperature Variations. Int. J. Pavement Eng..

[25] Pais J., Thives L., Pereira P., Pereira P., Pais J. (2024). Artificial Neural Network Models for the Wander Effect for Connected and Autonomous Vehicles to Minimize Pavement Damage. Proceedings of the 10th International Conference on Maintenance and Rehabilitation of Pavements, MAIREPAV 2024.

[26] Pais J., Pereira P., Thives L. (2023). Wander Effect on Pavement Performance for Application in Connected and Autonomous Vehicles. Infrastructures.

