# Supplementary material for: A Simple Model to Estimate the Increase in Pavement Life Due to the Traffic Wander for Application in Connected and Autonomous Vehicles
Source: Materials (Basel). 2025 Jun 3;18(11):2609. doi: 10.3390/ma18112609 (PMC12156044; doi:10.3390/ma18112609)
Supplement: Supplementary file 1 [file materials-18-02609-s001.zip › materials-3658047-supplementary.pdf]

Supplementary Information

A Simple Model to Estimate the Increase in Pavement Life Due to the Traffic Wander for Application in Connected and Autonomous Vehicles

Beata Gajewska [1,*], Marcin Gajewski [2], Jorge Pais [3,*] and Liseane Thives [4]

Table S1. Relative pavement life for cracking and rutting.

		Normal distribution										Uniform distribution									
		Variation of pavement life (cracking)					Variation of pavement life (rutting)					Variation of pavement life (cracking)					Variation of pavement life (rutting)				
h_{as}	E_{sub}	w=0.2	w=0.3	w=0.4	w=0.5	w=0.6	w=0.2	w=0.3	w=0.4	w=0.5	w=0.6	w=0.2	w=0.3	w=0.4	w=0.5	w=0.6	w=0.2	w=0.3	w=0.4	w=0.5	w=0.6
10	60	1.156	1.208	1.234	1.307	1.409	1.031	1.078	1.150	1.242	1.350	1.147	1.377	1.755	2.163	2.574	1.141	1.365	1.685	2.055	2.440
15	60	1.062	1.073	1.115	1.188	1.283	1.033	1.078	1.142	1.224	1.319	1.066	1.268	1.590	1.950	2.318	1.137	1.326	1.595	1.915	2.259
20	60	1.023	1.042	1.092	1.166	1.259	1.031	1.070	1.127	1.198	1.282	1.061	1.251	1.545	1.882	2.232	1.122	1.283	1.509	1.783	2.085
25	60	1.009	1.039	1.094	1.169	1.260	1.029	1.065	1.116	1.180	1.254	1.075	1.257	1.531	1.850	2.187	1.112	1.252	1.448	1.687	1.954
10	80	1.188	1.251	1.274	1.345	1.448	1.027	1.073	1.144	1.237	1.345	1.173	1.409	1.801	2.221	2.644	1.133	1.360	1.688	2.063	2.452
15	80	1.074	1.089	1.130	1.204	1.301	1.033	1.079	1.145	1.230	1.329	1.076	1.285	1.618	1.988	2.364	1.139	1.338	1.621	1.955	2.312
20	80	1.028	1.046	1.096	1.172	1.266	1.031	1.073	1.132	1.208	1.295	1.063	1.259	1.563	1.908	2.265	1.128	1.298	1.540	1.831	2.149
25	80	1.009	1.040	1.095	1.173	1.266	1.031	1.070	1.124	1.191	1.270	1.076	1.265	1.548	1.877	2.222	1.120	1.270	1.481	1.737	2.023
10	100	1.201	1.289	1.309	1.378	1.481	1.023	1.067	1.137	1.230	1.338	1.196	1.435	1.839	2.269	2.701	1.124	1.353	1.685	2.062	2.452
15	100	1.087	1.107	1.147	1.222	1.320	1.033	1.079	1.147	1.234	1.336	1.088	1.302	1.646	2.024	2.407	1.141	1.346	1.639	1.983	2.349
20	100	1.032	1.049	1.099	1.176	1.272	1.033	1.076	1.138	1.216	1.307	1.065	1.265	1.576	1.927	2.289	1.133	1.312	1.566	1.870	2.200
25	100	1.009	1.040	1.096	1.175	1.270	1.031	1.072	1.128	1.199	1.281	1.076	1.270	1.561	1.896	2.247	1.124	1.283	1.506	1.775	2.074
